# Effect of Stacking Sequence on Mechanical Properties and Microstructural Features within Al/Cu Laminates

**DOI:** 10.3390/ma16196555

**Published:** 2023-10-04

**Authors:** Lenka Kunčická, Radim Kocich

**Affiliations:** 1 Faculty of Materials Science and Technology, VŠB—Technical University of Ostrava, 17. Listopadu 2172/15, 70800 Ostrava, Czech Republic; 2 Institute of Physics of Materials, Czech Academy of Sciences, Žižkova 22, 61600 Brno, Czech Republic

**Keywords:** rotary swaging, clad composite, residual stress, microstructure

## Abstract

The study presents a method to prepare Al/Cu laminated conductors featuring two different stacking sequences using rotary swaging, a method of intensive plastic deformation. The primary focus of the work was to perform detailed characterization of the effects of room temperature swaging on the development of microstructures, including the Al/Cu interfaces, and internal misorientations pointed to the presence of residual stress within the laminates. The results revealed that both the Al and Cu components of the final laminates with 5 mm in diameter featured fine, more or less equiaxed, grains with no dominating preferential texture orientations (the maximum observed texture intensity was 2.3 × random for the Cu components of both the laminates). This fact points to the development of dynamic restoration processes during swaging. The analyses of misorientations within the grains showed that residual stress was locally present primarily in the Cu components. The Al components did not feature a substantial presence of misorientations, which confirms the dynamic recrystallization. Tensile testing revealed that the laminates with both the designed stacking sequences exhibited comparable UTS (ultimate tensile strength) of almost 280 MPa. However, notable differences were observed with regard to the plasticity (~3.5% compared to less than 1%). The laminate consisting of Al sheath and Cu wires exhibited very low plasticity as a result of significant work hardening of Al; this hypothesis was also confirmed with microhardness measurements. Observations of the interfaces confirmed satisfactory bonding of both the metallic components.

## 1. Introduction

Research in virtually every industrial field has led to the introduction of numerous innovative components, as well as a variety of modern materials, such as alloys prepared by methods of additive manufacturing (e.g., selective laser melting [1,2], direct energy deposition [3,4], plasma arc melting [5,6], etc.), (pseudo)alloys fabricated via powder metallurgy [7,8], hybrid materials [9,10], functionally graded materials [11,12], high entropy alloys (HEAs) [13,14], or composites [15,16,17]. Composites can be of various types and can consist of several materials (e.g., metals [18], ceramics [19], polymers [20], or their variations, such as metallic glasses with polymers [21], polymers with nanoparticles [22], or ceramics with polymers [23]). Metallic composites combine several metals within a single material which then features enhanced properties, when compared to the individual metallic components. The most prominent are the Metal Matrix Composites (MMCs). They benefit from the advantageous properties of both the metal forming the matrix and dispersed reinforcing particles/fibers, and thus, they are applicable even for demanding applications, such marine [24] or aviation [25]. Such materials can be tailored to specific applications; for example, composites reinforced with long fibers are especially favorable for applications in which anisotropy of mechanical and utility properties is required, such as in the automotive industry [26,27].

Clad and laminated composites featuring individual layers of metals, each introducing characteristic properties, are also popular. Laminates consisting of metallic layers are typically produced by methods based on welding, such as laser welding [28], laser beam welding [29], or explosive welding [30]. Nevertheless, irrespective of the composite type, phase distribution and grain size are among the primary factors affecting its properties, and welding and similar methods involving high processing temperatures can introduce local structural modifications (grain growth, undesired precipitation of second phases, etc.), as well as formation of intermetallic phases [31]. Possible development of intermetallics on the interfaces is one of the greatest disadvantages of laminated composites [32], as they are typically brittle and can contribute to debonding of the layers and consequently decrease the mechanical properties and lifetime of the laminate [33]. On the other hand, laminates can also be manufactured at room temperature using methods of (intensive and severe) plastic deformations. Such methods enable simultaneous achievement of high-quality bonding of the individual layers and formation of UFG (ultra-fine-grained) structure [34]. Therefore, they provide an advantageous solution for production of multi-component laminates under cold conditions [35].

Among the most widely researched laminates, layered composites are those consisting of Al and Cu [36,37]. Al is favored not only for its advantageous combination of reasonable strength and low density, but also for its sufficiently high electric conductivity, while Cu is added primarily for its excellent electric conductivity [38]. Furthermore, both the metals have wide applicability in transportation [39], automotive [40], electrotechnics [41], and numerous other commercial and industrial fields. However, the disadvantage is that they have the tendency to form various intermetallic compounds [42], i.e., to develop intermetallics at mutual interfaces, especially when processed at elevated temperatures [43]. For the Al/Cu laminates, performing a post-process heat treatment at a temperature of ~300–350 °C was reported to favorably support structural restoration and consequent grain refinement within both the metallic components. Nevertheless, it also imparts the development of intermetallic phases, which limits the possibilities of post-processing for such laminates [44]. Due to this reason, designing an optimized procedure for production of Al/Cu laminates, which would reduce/eliminate the undesirable development of brittle intermetallic phases, is among the main aims of the contemporary research.

Methods of intensive and severe plastic deformations (IPD and SPD) are advantageous for production of various composites, since they can be performed at low/ambient temperatures [45]. Among the typically researched and applied SPD methods are equal channel angular pressing (ECAP) [46,47] and its modifications (twist channel angular pressing (TCAP) [48,49], twist channel multi angular pressing (TCMAP) [50], non-ECAP (NECAP) [51,52], ECAP with partial back pressure (ECAP-PBP) [53,54], etc.), methods combining torsion and extrusion [55,56], or high pressure torsion (HPT) [57,58]. Most of the SPD methods are designed to process samples of relatively small dimensions (except ECAP-Conform [59,60] and a few other continuous methods, such as continuous confined strip shearing (C2S2) [61,62], or single roll angular rolling (SRAR) [63,64]). On the contrary, the intensive plastic deformation method of rotary swaging (RS) [65,66] is continuous and is highly effective for grain refinement, similar to SPD methods. RS enhances the structures and properties of the processed materials via imposing intensive shear strain, while enabling the shaping of the product to desired final dimensions. It is a versatile technology, which is also applied in the industry (primarily in the automotive industry) to manufacture various solid and/or hollow parts. During the process, repeated action of rotating dies incrementally affects the processed workpiece with high shear strain and compressive radial forces, and the mutual effect of which introduces favorable combinations of shear and compressive strains, enabling to avoid large stress gradients and supporting progressive grain refinement and resulting in gradual improvement of mechanical and utility properties.

This study presents the method of preparation of laminated Al/Cu conductors via room temperature RS. The laminates featured two different designs, i.e., two individual stacking sequences (Al sheath with Cu wires and Cu sheath with Al wires). The primary aim was to characterize the effects of RS on (sub)structure development and evolution of internal misorientations, i.e., residual stress, within the final laminates swaged to the diameter of 5 mm.

## 2. Materials and Methods

### 2.1. Experimental Material

**Figure 1:**
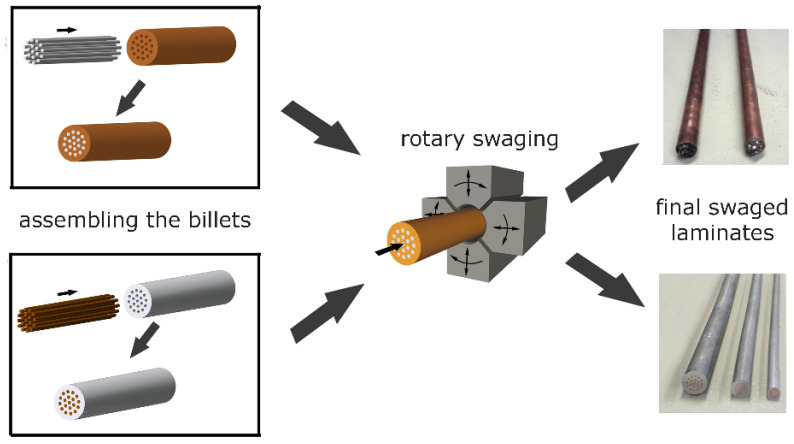
Schematic depiction of manufacturing process of the laminates.

The original metals used to assemble the laminated billets were commercially pure (CP) electro-conductive Cu (with additions of 0.002 wt.% O, 0.015 wt.% P, and 0.002 wt.% Zn) and CP electro-conductive Al (with additions of 0.20 wt.% Si, 0.25 wt.% Fe, and 0.05 wt.% Cu). The laminates of both the stacking sequences initially consisted of a sheath with 30 mm diameter, and 19 inserted wires with 3 mm diameter, each. The stacking sequence *A* was designed to consist of Al sheath and Cu wires, while the stacking sequence *B* consisted of Cu sheath and Al wires. The assembled clad billets were gradually swaged at room temperature from the initial diameter of 30 mm to laminated wires with the final diameter of 5 mm. Schematic depiction of the manufacturing process can be seen in Figure 1. The final swaging degree for both the laminates was 3.6 (calculated using Equation (1)),

(1) $$\phi =\ln\left(\frac{S_{0}}{S_{n}}\right)$$

where *S*_0_ and *S_n_* are cross-section areas of the laminate at input and output of the dies.

### 2.2. Investigations

Analyses of microstructures of both the swaged 5 mm laminates were performed with scanning electron microscopy (SEM), as well as transmission electron microscopy (TEM). Tescan Lyra 3 FIB/SEM microscope (Tescan Tescan Orsay Holding a.s., Brno, Czech Republic) equipped with a NordlysNano detector (OXFORD Instruments, Abingdon-on-Thames, Great Britain) was used to perform electron back-scattered diffraction (EBSD) observations. Samples for SEM analyses were prepared from transversally cut sections through the swaged laminates (cutting was performed using electrical discharge machining, EDM). The samples were further ground on SiC papers, and finally electrolytically polished. The EBSD scans were performed with the step of 0.25 µm. The scanned data was evaluated with the help of AZTec software (Version 6.1, OXFORD Instruments, Abingdon-on-Thames, Great Britain) and ATEX software (Version 1.32, LEM3 Laboratories, Metz, France) [67]. During the evaluations, the values of 5° and 15° were considered as the limits for low- and high-angle grain boundaries (LAGBs and HAGBs), respectively. Texture orientations were evaluated with the maximum deviation of 10°.

Samples for detailed substructure analyses via TEM were prepared from transversally cut sections, which were ground manually and finished with electrolytical polishing. Observations were performed using a Jeol 2100F TEM device (Akishima, Tokyo, Japan) operating at 200 kV. For both the laminate designs, all the micro- and substructure observations were performed in radial locations corresponding to the peripheral layers of inserted wires.

Vickers microhardness measurements of the components of the laminates were performed using a Zwick Roell DuraScan 70 G5 machine (Zwick Roell CZ s.r.o., Brno, Czech Republic). The average HV02 values for the sheaths of the laminates were calculated from ten individual indents executed across the particular laminate cross-section (excluding the wires), whereas the average HV02 values for the wires of each laminate were calculated from ten individual indents executed throughout all the wires across the cross-section of the particular laminate. Similarly, the average HV02 values for the original metallic components were calculated from ten indents executed randomly across their cross-sections. A Zwick/Roell testing machine was also used to perform tensile tests of the swaged laminates. The tensile tests were performed with the use of laminated samples having the length of 100 mm, and the testing speed was 10^−3^ s^−1^. The testing was executed according to the DIN EN ISO 6892-1 standard [68].

## 3. Results and Discussion

### 3.1. Deformation Behavior

**Figure 2:**
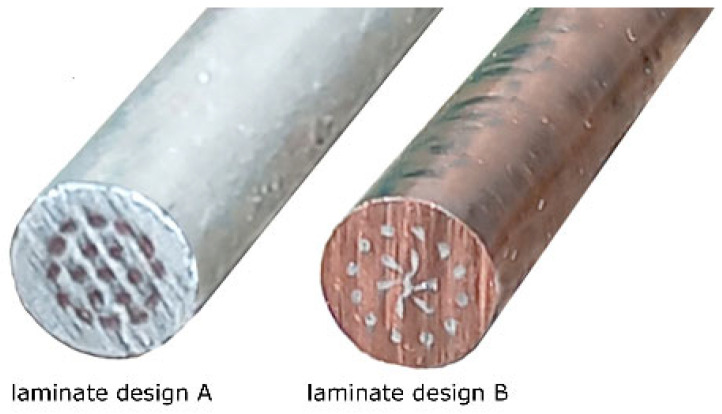
Final 5 mm swaged laminates of design A (Al sheath), and design B (Cu sheath).

Figure 2 shows photos of the final swaged laminates of both the designs. The figure clearly shows that the behavior of the inserted wires was different within both the laminates, especially with regard to the axial wire and the internal layer of wires (see that these Al wires within laminate B were deformed more than those within laminate A). This can be primarily correlated with the acquired results of mechanical properties of both the laminates and the character of the swaging process, i.e., plastic flow of the material during swaging, as further discussed in Section 4.

### 3.2. Microstructure Analyses

**Figure 3:**
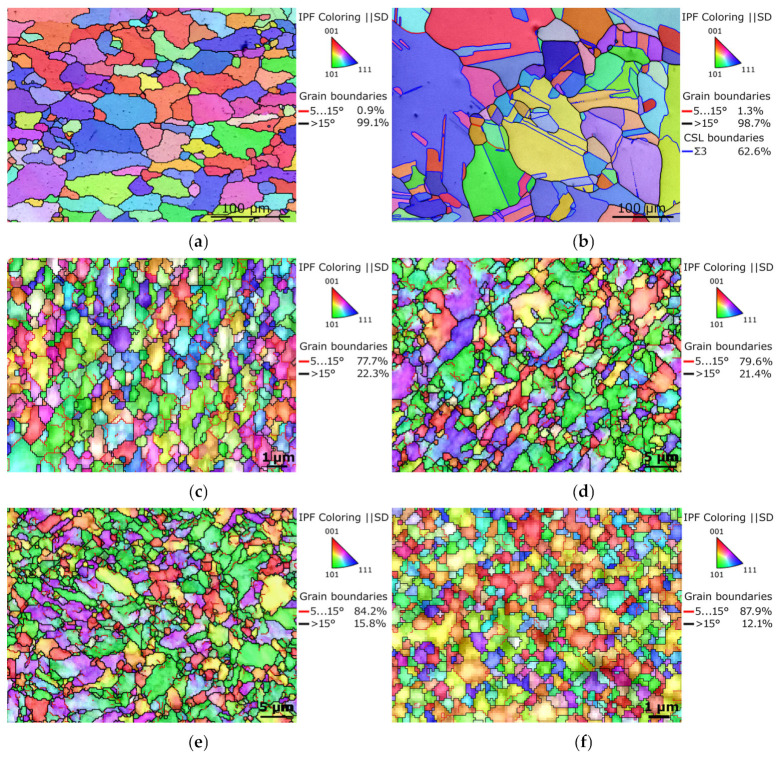
OIM images depicting microstructures of original Al (**a**); original Cu (**b**); Al sheath of laminate A (**c**); Cu wire of laminate A (**d**); Cu sheath of laminate B (**e**); and Al wire of laminate B (**f**).

The orientation image maps (OIM), depicting the microstructures of the metallic components from which the billets were assembled, are shown in Figure 3a,b. The maps also depict the LAGB and HAGB fractions, as well as <111> 60°, i.e., Ʃ3 coincidence site lattice (CSL), and twin boundaries (for the Cu). The microstructures of both the original metals evidently featured coarse recrystallized grains having no prevailing preferential orientation. The original Al featured the majority of HAGBs, and so did the original Cu, which also exhibited a substantial presence of recrystallization twins (62.6% of HAGBs were of the Ʃ3 type). The average grain size, evaluated via the maximum Feret diameter parameter [69], was 63.9 µm for the Al, and 36.3 µm for the Cu.

Figure 3c,d show OIM images of the microstructures of the Al sheath and Cu wire of laminate A, and Figure 3e,f depict OIM images of the microstructures of the Cu sheath and Al wire of laminate B (the grain colors are depicted by ||*z* axis, i.e., shear direction—SD). The results of the microstructure observations revealed that the room temperature swaging process led to significant grain refinement for both the metals within both the swaged laminates. However, the grains within the Al components of the swaged laminates were generally smaller than within the Cu ones. The average grain size for the Al sheath of laminate A was 1.1 µm, while for the Cu wires, it was 3.7 µm. For laminate B, the average grain size for the Cu sheath was 2.9 µm, while the value for the grains of the Al wires was 1.7 µm. The OIM images show that especially the Al components of both the laminates featured fine, more of less equiaxed grains, and the sizes of which reached almost at the ultra-fine (UF) scale.

The grain boundary analyses revealed that both the components of both the laminate designs exhibited prevailing volume fractions of HAGBs; these fractions were 77.7% for the Al and 79.6% for the Cu components of laminate A, and 84.2% for the Cu and 87.9% for the Al of laminate B.

### 3.3. Texture

**Figure 4:**
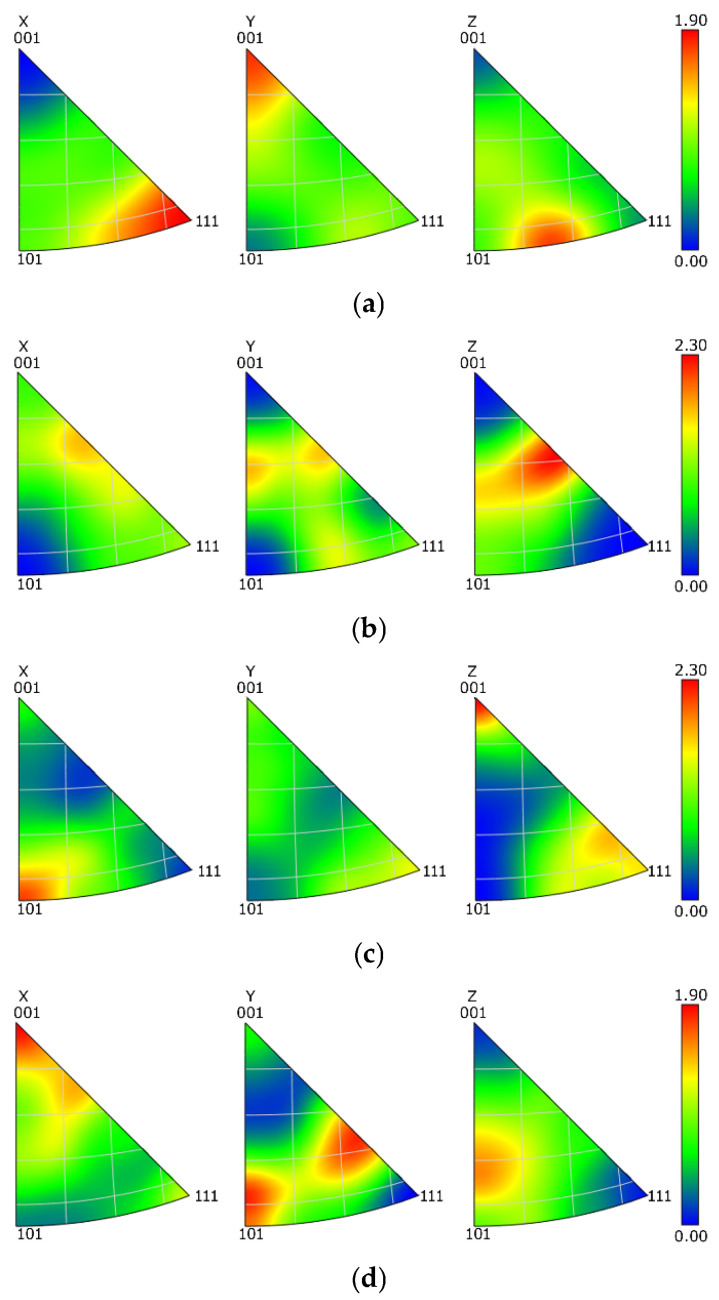
IPF depicting textures within Al sheath of laminate A (**a**); Cu wire of laminate A (**b**); Cu sheath of laminate B (**c**); and Al wire of laminate B (**d**).

The mapping of grains orientations within the components of the swaged laminates (Figure 3c–f) showed that the grains did not feature any prevailing preferential orientation in general, i.e., the colors in the OIM images were more or less random. In order to document this statement with a greater reliability, texture analyses via inverse pole figures (IPF) were performed. The IPF images depicting preferential texture fibers within the Al and Cu components of both the laminates are shown in Figure 4a–d. As can be seen, the texture intensities were low, which corresponds to the above-presented OIM data; the maximum intensity of up to 2.3 × random was observed for both the Cu components of the laminates, while the maximum texture intensity of the Al components of both the laminates was lower than 2 × random. The observed preferential texture orientations differed slightly, most probably in accordance with the level of occurring restoration processes, which will be further discussed in Section 4.

### 3.4. Internal Misorientations

**Figure 5:**
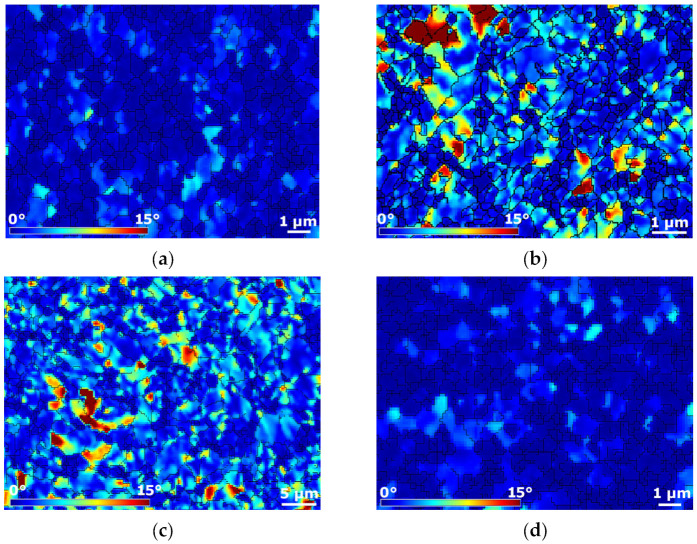
Internal misorientations pointing to presence of residual stress within Al sheath of laminate A (**a**); Cu wire of laminate A (**b**); Cu sheath of laminate B (**c**); and Al wire of laminate B (**d**).

The internal grains misorientations in the scale from 0° to 15° pointing to the presence of residual stress within the microstructures of Al and Cu components of the swaged laminates are shown in Figure 5a–d (microstructure locations correspond to those depicted in Figure 3c–f). The results of the analyses revealed that the presence of internal misorientations within the microstructure of Al sheath of laminate A was scarce (Figure 5a), and so was their presence within the Al wires of laminate B (Figure 5d). On the other hand, a significant portion of the grains within the microstructures of the Cu components of both the swaged laminates exhibited the presence of (high) misorientations pointing to the occurrence of residual stress, which was most probably related to the plastic flow during processing, and development of substructure within these grains, as further discussed.

### 3.5. Mechanical Properties

The mechanical properties of the swaged laminates, as well as of the original metals, were at first assessed with Vickers microhardness measurements. The acquired values are summarized in Table 1. The results of the measurements of the swaged laminates showed that the average microhardness value increased for both the components of both the laminates. Evidently, the HV02 values for the Cu components of both the laminates were comparable, whereas the average HV02 value of the Al component of laminate A was substantially higher than that acquired for the Al component of laminate B. The table also shows that the standard deviation values were higher for the Cu components of the swaged laminates than for the Al ones.

**Figure 6:**
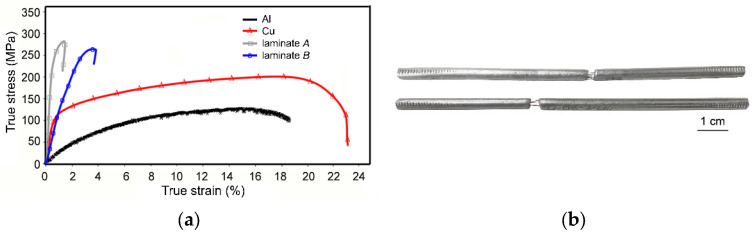
Stress–strain curves acquired from tensile tests for both 5 mm swaged laminates (**a**); specimens of laminate A after testing (**b**).

The stress–strain curves originating from the tensile tests of the original metallic components, as well as both the swaged laminates, are shown in Figure 6a. As can be seen from the acquired results, both the laminate designs exhibited comparable UTS values, but the plasticity of the laminates differed. The elongation to failure for laminate B, featuring a dominant volume fraction of Cu, was ~3.5%. On the other hand, for laminate A, featuring a dominant volume fraction of Al, the elongation to failure was less than 1%. Figure 6a shows that the stress–strain curve acquired for laminate A featured a steep increase from the very beginning of loading. In other words, laminate A exhibited substantial work hardening. This is documented also by Figure 6b showing the specimens of laminate A after testing; the specimens evidently show very negligible necking before fracture. Laminate B also exhibited work hardening, as can be seen from the rapidly increasing true stress, but its intensity was not as high as for laminate A.

### 3.6. Interfaces

In order to document the quality of bonding of the individual metallic components within the laminates after swaging, the interface between the metals was investigated via both SEM and TEM. Considering the above-presented results of testing of mechanical properties of the laminates and their deformation behavior, the interfaces within laminate design B were primarily examined (the individual metallic components of this laminate exhibited greater differences in the mechanical properties, see Table 1). Therefore, stress shielding and cracking at the interfaces were more likely to occur within laminate B.

**Figure 7:**
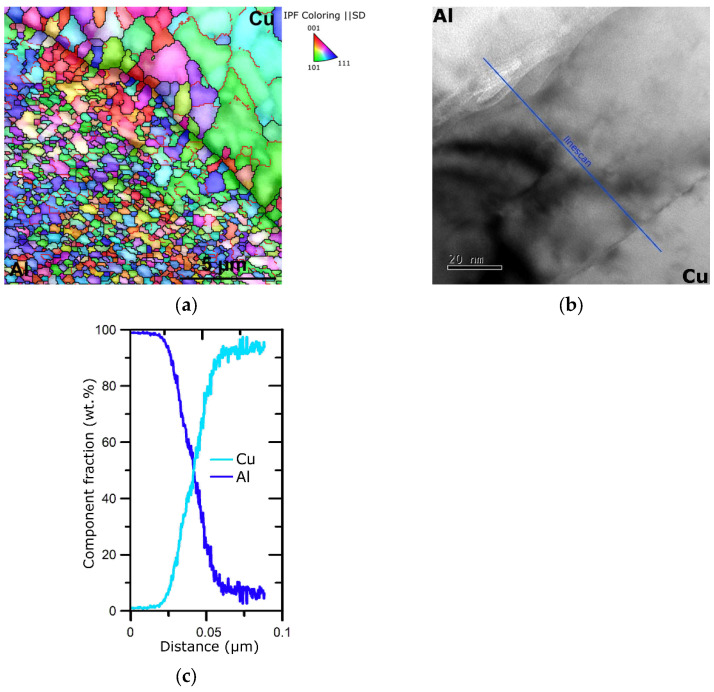
OIM image depicting Al wire/Cu sheath interface of laminate design B (**a**); TEM scan of the interface depicting high-quality mechanical bonding of Al and Cu (**b**); chemical composition of interface measured along the linescan depicted in Figure 6b (**c**).

Figure 7a depicts the OIM image of the Al/Cu interface within laminate B, whereas Figure 7b shows a detailed TEM image of such an interface. The OIM image documents that the individual metallic components of the swaged laminate were well bonded at mutual interfaces, which did not exhibit any voids or cracks. It also confirms the above-mentioned data acquired from microstructure analyses, i.e., both the metallic components featured heavily refined grains (especially the Al one), and neither the Al nor the Cu components exhibited a dominant texture orientation. The detailed TEM scan of the Al/Cu interface shows that there was a transition zone featuring a mix of both the metals despite the fact that the swaging was performed at room temperature. The chemical composition of the transition zone at the Al/Cu interface, measured along the linescan depicted in Figure 7b, can be seen in Figure 7c. However, this transition zone was only about 60 nm wide and did not negatively influence the mechanical behavior of the laminate with regard to the possible development of cracks (as also confirmed with the OIM image in Figure 6a, depicting a larger region of the mutual interface).

## 4. Discussion

Practically, all the methods of intensive and severe plastic deformations can be applied to prepare various kinds of composites and laminates, but the substructure development of multi-phase materials during processing is generally different from the substructure development of the single-phase ones. This also involves increasing dislocation density resulting in formation of dislocation cells, the size of which is typically in a reciprocal proportion to the imposed strain (i.e., their size typically decreases with increasing strain). With increasing imposed strain, the substructure featuring LAGBs continuously transforms into a homogeneous fine-grained structure featuring HAGBs [70]. On the other hand, for multi-phase materials deformed via intensive plastic deformation, the decreasing grain size is not in any direct proportion to the imposed strain. Despite the fact that decreasing size/thickness of the reinforcing components or layers provokes decrease in the grain size during plastic deformation, accumulation of critical imposed strain can result in the formation of saturated solid solution (amorphous structure) [71,72,73]. Based on the interactions of the individual composite components during intensive plastic deformation, the achievable final grain size is generally smaller than that for single-phase materials [74]. In other words, processing of composites and laminates via intensive plastic deformation introduces (repeated) development of structural restoration processes even when processed at ambient temperatures, which not only result in significant grain refinement, but also in relaxation and homogenization of possible residual stress, and enhancement of properties/lifetime of the final product.

For the herein investigated swaged laminates, the grains within the Al components were evidently smaller than within the Cu components for both the laminate designs. This phenomenon was primarily based on the fact that the activation energy needed to promote relaxation processes within Al is generally lower than the activation energy for Cu [38]. The substantial grain refinement observed for the structures of both the metallic components of the swaged laminates was particularly introduced by the intensive plastic deformation method of rotary swaging. This method gradually imparts small increments of shear strain, by which it supports repeated dynamic recrystallization [17]. The occurrence of dynamic recrystallization introduced by the high imposed shear strain was confirmed not only by the severely refined grains, but also by the grain boundary analyses, and the analyses of internal grains misorientations documented that the metallic components (especially Al) featured more or less relaxed microstructures. Recrystallization was even supported by the predominantly compressive stress character of the swaging process, and this factor also contributed to the satisfactory bonding of both the metals, even when swaged at room temperature. As documented in Figure 6a,b, the Al/Cu mutual interface featured just a very thin transition zone consisting of an Al-Cu solid solution. Therefore, no brittle intermetallic phases, which would induce the possible development of cracks and thus deteriorate the mechanical properties and lifetime of the laminates, were observed.

Although the average HV02 microhardness values of the Cu components were comparable for both the laminate designs, the average HV02 values of the Al components differed significantly. Also, the standard deviations for the HV02 values were higher for the Cu components, while for the Al ones, they were more or less negligible. This can be explained by the (localized) presence of internal misorientations, i.e., accumulated residual stress, within the structures. Although the occurrence of misorientations within the Al components of both the laminates was scarce, misorientations were locally present within the structures of the Cu components of the laminates, which also was most probably caused the local deviations from the average microhardness values. The fact that HV02 of the Al sheath of laminate A was higher than that of the Al wires of laminate B can be explained by the mutual effect of the following factors: the shear strain primarily affects peripheral regions of the workpiece during swaging. As a consequence, a considerable amount of shear strain was imposed during swaging into the Al sheath for laminate A. Therefore, also considering the intrinsic properties of Al (especially its stacking fault energy [38]), the maximum work hardening ability of the Al of laminate A was reached during swaging. This hypothesis was supported not only by its very fine, almost UF, grain size, but also by the results of tensile testing. The relatively high ultimate tensile strength (UTS) achieved for laminate A can be attributed to the mentioned achieved maximum work hardening ability of Al. On the other hand, the relatively high UTS achieved for laminate B can primarily be attributed to the high-volume fraction of Cu.

The presence of Cu within both the composites contributed not only to their advantageous strength, but also provided favorable plasticity [75], which was observed especially for laminate B. In other words, the relatively high plasticity of laminate B can primarily be attributed to the major volume fraction of Cu (the plasticity of laminate A was exhausted due to the major content of the work hardened Al). The differences in plasticity of the laminates were also directly related to their plastic flows during swaging, and thus to the deformation behaviors of their metallic components. As documented by Figure 2, the plastic flows of the inserted wires, especially in the internal layers, differed for both the laminates. For laminate design A, the imposed strain, i.e., imparted energy, tended to accumulate in the Al sheath, which caused work hardening and exhaustion of plasticity. Based on this factor, the plastic flow of the Al sheath was predominantly in the axial direction during swaging, and the vortex-like plastic flow (induced by the rotary movement of the swaging dies) was suppressed. On the other hand, the plastic flow of laminate B, the sheath of which consisted of Cu featuring a higher plasticity, was not only in the axial direction, but also in the tangential direction. The effect of the vortex-like flow can particularly be seen in the axial region of the laminate, in which the fan-like appearance of the internal Al wires is evident. In other words, the higher plasticity of the Cu sheath (when compared to the Al sheath) enabled vortex-like flow of the material, which consequently resulted in a more substantial deformation of the cross-sections of the inserted wires. This unconventional effect of the imposed strain can primarily be attributed to two main phenomena. The first one was friction between the Cu sheath of laminate B and the swaging dies, whereas the second one was related to the temperature-dependent plastic flow of the material. As for the friction, the vector of plastic flow velocity in the surface and subsurface areas of laminate B caused the axial plastic flow to become dominant in these regions (as confirmed by the shapes of the inserted Al wires). In other words, the friction aggravated the tangential plastic flow in the mentioned areas, as documented by the negligible deformation of the cross-sections of the Al wires in the outer layer. On the contrary, the internal and axial regions of laminate B exhibited mixed character of plastic flow (as seen in Figure 2). Such a distribution of the imposed strain is enabled when the processed workpiece features areas with different plasticities. For the herein presented laminates, the temperature distribution during swaging could play a major role in this. The presented laminates were swaged at room temperature. However, a significant portion of the imposed strain is generally transformed into deformation heat [76]. As a consequence, internal regions of the presented laminates were defined by slightly elevated processing temperatures (also, the surface areas were quickly cooled by the cold swaging dies). This hypothesis is in agreement with the mentioned differences in the development of softening processes occurring in the Cu components of the laminates. The variations in temperature of the sheath (primarily the Cu featuring a higher plasticity) directly influenced character of the plastic flow, as confirmed by the cross-sectional shapes of the inserted wires. This factor also contributed to the grain refinement observed within the Al wires of laminate B; the average grain size within these wires was more or less comparable to that acquired for the Al sheath of laminate A, despite the fact that the direct effect of the imposed strain on these wires was lower when compared to the Al sheath.

## 5. Conclusions

The presented study investigated the effects of room temperature rotary swaging on Al/Cu laminates of two designs—Al sheath and Cu wires and Cu sheath and Al wires. The results of the study (provided as bullet points) can be summarized as follows:

- Mechanical bonding of the layers via intensive plastic deformation was successful;
- The individual metallic components featured heavily refined grains, regardless of the laminate design;
- Al components of both the laminate designs featured equiaxed, almost ultra-fine recrystallized grains, no dominant preferential texture, and negligible presence of internal misorientations;
- Cu components of both the laminate designs featured grains with average sizes of ~3 µm, no dominant texture, and local presence of internal misorientations;
- Notable differences were observed for the mechanical properties. The laminate with Al sheath exhibited plasticity lower than 1% due to the significant work hardening of Al (microhardness ~55 HV02), while the laminate with Cu sheath featured plasticity of ~3.5%, primarily as the content of work-hardened Al was lower.

The study confirmed that the rotary swaging method is suitable for the fabrication of such Al/Cu laminates.

## Figures and Tables

**Table 1:** Average Vickers microhardness values for metallic components of swaged laminates.

Material	Metal	Avg. Microhardness (HV02)
original CP	Al	84.8 (±1.4)
original CP	Cu	25.1 (±1.7)
laminate design	metallic component	
A	Cu wires	107.6 (±4.9)
A	Al sheath	54.8 (±1.2)
B	Al wires	34.3 (±0.9)
B	Cu sheath	108.8 (±6.2)

## Data Availability

The original data supporting the research are not publicly available, but a portion of the data that is not confidential is available on request from the corresponding author.
